# Kinetically Consistent Coarse Graining Using Kernel-Based Extended Dynamic Mode Decomposition

**DOI:** 10.1021/acs.jctc.5c00479

**Published:** 2025-07-18

**Authors:** Vahid Nateghi, Feliks Nüske

**Affiliations:** 28307 Max-Planck-Institute for Dynamics of Complex Technical Systems, Magdeburg 39106, Germany

## Abstract

In this paper, we show how kernel-based models for the Koopman generator—the gEDMD method—can be used to identify coarse-grained dynamics on reduced variables, which retain the slowest transition time scales of the original dynamics. The centerpiece of this study is a learning method to identify an effective diffusion in coarse-grained space, which is similar in spirit to the force matching method. By leveraging the gEDMD model for the Koopman generator, the kinetic accuracy of the CG model can be evaluated. By combining this method with a suitable learning method for the effective free energy, such as force matching, a complete model for the effective dynamics can be inferred. Using a two-dimensional model system and molecular dynamics simulation data of alanine dipeptide and the Chignolin mini-protein, we demonstrate that the proposed method successfully and robustly recovers the essential kinetic and also thermodynamic properties of the full model. The parameters of the method can be determined using standard model validation techniques.

## Graphical abstract

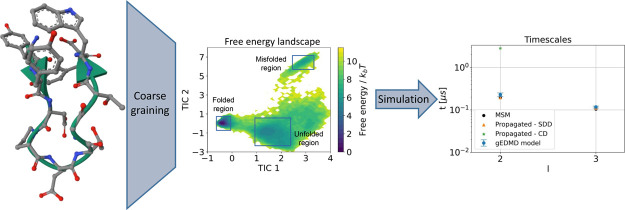

## 1 Introduction

Stochastic simulations of large-scale dynamical systems are widely used to model the behavior of complex systems, with applications in computational physics, chemistry, materials science, and engineering. Many examples of such systems are high dimensional and subject to meta-stability, which means the system remains trapped in a set of geometrically similar configurations, while transitions to another such state are extremely rare. As a consequence, it becomes necessary to produce very long simulations in order to make statistically robust predictions. A prime example are atomistic molecular dynamics simulations (MD)^1^ of macro-molecules, where meta-stability is typically caused by high energetic barriers separating deep potential energy minima.^2^ As a result, it requires specialized high-performance computing facilities to reach the required simulation times, or it may just not be feasible at all.^3^

Coarse graining (CG) describes the process of replacing the original dynamical system by a surrogate model on a (much) lower-dimensional space of descriptors,^4,5^ in such a way that certain properties of the original dynamics are preserved. CG models can enable scientists to achieve much longer simulation times because of the reduced computational cost, while maintaining predictive capabilities of the full-order model. Setting up a CG model typically requires the following steps: first, the choice of a linear or nonlinear mapping (CG map) from full state space to a lower-dimensional space, where the latter serves as the state space of the surrogate model. Second, definition of a parametric model class for the surrogate dynamics. Finally, fitting the parameters of the selected model class using available data.

The first step is crucial to the CG model’s success, and has been a very active area of research for a long time, see refs 6–8 for reviews on this topic. Traditionally, coarse grained coordinates have been based on molecular structure, e.g., by considering only alpha-carbons or reduced atom representations. More recently, CG projections into less interpretable and nonlinear spaces have also been considered, such as the latent space of a neural network transformation.^9,10^ The selection and quality of the CG coordinate is not the central aspect of this study, we focus on model selection and parameter fitting instead. Therefore, we only show examples of low-dimensional CG coordinates that have already been validated, and that are not directly transferable. The problem of learning high-dimensional and fully transferrable CG models along with their collective variables is left for future studies.

CG models have often been parametrized using physically intuitive functional forms for the coarse-grained energy. More recently, much more general functional forms have been used for the CG parameters, which are then approximated by powerful model classes, such as deep neural networks or reproducing kernels,^11–13^ which is the approach we follow in this paper. We study CG for reversible stochastic differential equations (SDE) with a Boltzmann-type invariant distribution, such as Langevin dynamics. Theoretical frameworks to CG modeling are typically based on projections of dynamical evolution operators. This includes the Mori-Zwanzig formalism,^14,15^ as well as the approaches by Gyöngy,^16^ Legoll and Lelièvre,^17^ and the averaging/homogenization framework.^18^ We follow Legoll and Lelièvre’s projection method, which means to parametrize the coarse-grained model as a reversible SDE, disregarding memory terms. The theoretical properties of this approach have been studied to quite some extent in the literature.^19–21^

The success of machine learning (ML) in recent years has led to the development of many powerful learning schemes for the parameters of a CG model, see ref 22 for a comprehensive overview. Examples are free energy learning,^23^ and force matching,^24^ among others. Many of these learning methods are geared toward ensuring *thermodynamic consistency*, which means that the marginalized Boltzmann distribution in CG space is preserved. Ensuring faithful reproduction of kinetic properties, such as time-correlation functions or transition time scales, is a much less developed topic. Besides the theoretical contributions noted above, several authors have focused on preserving specific dynamical observables or time-dependent distributions by incorporating these quantities into the learning process.^25–28^ Furthermore, several recent studies have considered integrated learning frameworks for CG coordinates and associated dynamics geared toward preserving transition rates, using autoencoders,^9^ normalizing flows^10^ or diffusion maps.^29^

In this paper, we combine learning of a coarse-grained SDE with Koopman operator models in order to recover implied transition time scales^30^ associated with meta stable states. Transition time scales are derived from the leading spectrum of the Koopman generator.^31–34^ This connection has been at the heart of the Markov state modeling (MSM) approach^30,35,36^ and many important developments based on it.^37–39^ The *spectral matching* approach,^40^ later formalized in ref 41, was the first to make use of this connection, by parametrizing the CG model as a linear expansion of fixed basis functions, and then solving a regression problem to recover the eigenvalues of the Koopman generator. The generator matrix can be estimated by a data-driven algorithm called generator EDMD (gEDMD).^41^

We significantly improve on the idea of leveraging the Koopman generator for the identification of coarse grained models in the following ways:

- Based on the projection approach, we formulate a stand-alone learning problem for the effective diffusion of a coarse-grained SDE. This formulation is analogous to the force matching approach for the coarse-grained energy. Just as force matching relies on measurements of the local mean force, our approach rests on a similar quantity called local diffusion. Combined with a suitable estimate for the free energy, the learned effective diffusion provides a closed-form expression for the CG dynamics.
- We suggest to parametrize the diffusion by a basis of random Fourier features,^42^ which form a widely used approximation technique for reproducing kernels. Random features offer a compromise between representational power and computational efficiency. The only hyper-parameters to be tuned are those of the kernel function. Conveniently, we show that the same random feature basis can be used to train a kinetic model for the Koopman generator. The method is robust to statistical noise and ill-conditioning as it is based on a whitened and truncated basis set.
- We show that gEDMD models can be leveraged to evaluate the kinetic consistency of the learned CG model on-the-fly by comparing its eigenvalues to those of the reference gEDMD matrix. Importantly, this assessment does not require simulations of the CG model.
- We show that kinetic and also thermodynamic consistency are achieved by the method using three test cases, a two-dimensional model system and molecular dynamics simulations of the alanine dipeptide and the Chignolin mini-protein. For the molecular systems, we learn a CG model corresponding to overdamped Langevin dynamics. The results show that for systems close enough to the overdamped limit, this approximation leads to a uniform rescaling of the slow time scales, which can be explicitly corrected for.

The structure of the paper is as follows: we introduce the required background on SDEs, coarse graining, and Koopman operator learning in Section 2. Our learning framework is then presented in Section 3, while the numerical examples follow in Section 4. Additional information on simulation details and model selection is given in the Supporting Information.

## 2 Theory

In this section, we provide the necessary background on stochastic dynamics, data-driven modeling, and Koopman spectral theory. The important notation used in the manuscript is summarized in Table 1.

**Table 1:** Overview of Notation

Symbol	Definition
*X*_*t*_	stochastic process
$K^{t}$	Koopman operator with lag time *t*
L	generator of the Koopman operator
**h**	reduced basis set from whitening transformation
**L̂**, **L̂**_*r*_	generator matrix and reduced generator matrix
σ_α_^ξ^	effective diffusion parametrized by α
**L̂**_α_^ξ^	effective generator matrix for diffusion with parameters α
*V*, *F*	potential and effective potential
*f*_lmf_^ξ^, *a*_loc_^ξ^	local mean force and local diffusion
*A* ·_|*i*,*j*_ *B*	contraction of dimensions *i* and *j* of arrays *A* and *B*

### 2.1 Stochastic Processes

We consider a dynamical system described by a stochastic differential equation (SDE)

$$dX_{t}=b(X_{t})dt+\sigma (X_{t})dW_{t}$$ 1

where $b(X_{t}):R^{d}\to R^{d}$ is the drift vector field, $\sigma (X_{t}):R^{d}\to R^{d\times d}$ is the diffusion field, and *W*_*t*_ is a d-dimensional Brownian motion. The diffusion covariance matrix is denoted as $a\in R^{d\times d}$ :

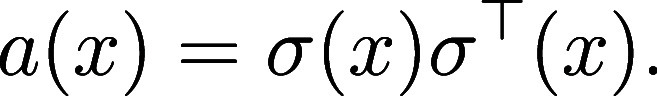

2
A standard example for eq 1, commonly used in molecular modeling, is overdamped Langevin dynamics

$$dX_{t}=-\frac{1}{\gamma }\nabla V(X_{t})dt+\sqrt{2\beta^{-1}\gamma^{-1}}dW_{t}$$ 3

where V:Ω→R is the potential energy, β = (*k*_B_ *T*)^−1^ and γ are constants corresponding to the inverse temperature and the friction, respectively. The invariant measure for *X*_*t*_ in eq 3 is the Boltzmann distribution μ ∝ exp­(−β*V*), and the dynamics are reversible with respect to μ. More generally, a reversible SDE with invariant measure μ ∝ exp­(−*V*) can be parametrized in terms of the generalized scalar potential 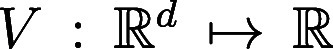, and the diffusion covariance *a*, as follows^43^

$$dX_{t}=\left[-\frac{1}{2}a(X_{t})\nabla V(X_{t})+\frac{1}{2}\nabla \cdot a(X_{t})\right]dt+\sigma (X_{t})dW_{t}$$ 4

We will only consider reversible SDEs in this paper, and make use of the parametrization in eq 4 when formulating learning methods.

### 2.2 Koopman Generator and Spectral Decomposition

Koopman theory^44,45^ lifts the dynamics in eq 1 into an infinite-dimensional space of observable functions to express the dynamics linearly. More precisely, the family of Koopman operators $K^{t}$ for stochastic dynamics is defined as

$$K^{t}\psi (x)=E^{x}[\psi (X_{t})]=E[\psi (X_{t})|X_{0}=x]$$ 5

where ψ is a real-valued observable of the system, and E[·] denotes the expected value. The associated infinitesimal generator L is the time-derivative of the expectation value, which can be written as a linear differential operator:

$$\begin{array}{rl} L\psi (x) & =b(x)\cdot \nabla \psi (x)+\frac{1}{2}a(x):\nabla^{2}\psi (x)\\ & =\overset{d}{\underset{i=1}{\sum }}b_{i}(x)\frac{\partial }{\partial x_{i}}\psi (x)+\frac{1}{2}\overset{d}{\underset{i,j=1}{\sum }}a_{ij}(x)\frac{\partial^{2}}{\partial x_{i}\partial x_{j}}\psi (x) \end{array}$$ 6

where *a* and *b* are the diffusion and drift terms defined above, ∇^2^[·] is the Hessian matrix of a function, and the colon: is a short-hand for the dot product between two matrices. For overdamped Langevin dynamics, eq 6 simplifies to

$$L\psi (x)=-\frac{1}{\gamma }\nabla V(x)\cdot \nabla \psi (x)+\frac{1}{\gamma \beta }\Delta \psi (x)$$

The key quantity of interest are the eigenvalues and eigenfunctions of the generator. The study of spectral components of the generator helps us identify the long-time dynamics of the system. In molecular dynamics, we expect to find a number of eigenvalues close to zero, followed by a spectral gap. These low-lying eigenvalues are indicating the number of metastable states of the system, which are the macro states the system stays in the longest.^31^ We write the eigenvalue problem for the generator as

$$-L\psi_{i}=\lambda_{i}\psi_{i}$$ 7

The eigenvalues λ_*i*_ of −L must be non-negative, and the lowest eigenvalue λ_1_ = 0 is nondegenerate:^46^ 0 = λ_1_ < λ_2_ ≤ λ_3_ ≤··· We also refer to the eigenvalues as rates, and to their reciprocals as implied time scales^30^

$$t_{i}=\frac{1}{\lambda_{i}}$$ 8

### 2.3 Coarse Graining and Projection

One of the main motivations of this work is to learn an SDE representing the full dynamics ([Disp-formula eq1]) on a coarse grained space. Coarse graining (CG) is realized by mapping the state space Ω onto a lower-dimensional space $\mathrm{\Omega ̂}\subset R^{d}$ by means of a smooth CG function ξ. We write ν ∝ exp­(−*F*) for the marginal distribution of the full-space invariant measure μ, where *F* is the free energy in the CG space.

To define dynamics in the CG space, we use the *conditional expectation operator*,^17,19^ also called *Zwanzig projector*:

$$P\psi \left(z\right)=E^{\mu }\left[\psi \left(x\right)|\xi \left(x\right)=z\right]$$ 9

where *z* is a position in CG space. This operator calculates the average of a function ψ over all *x* ∈Ω whose projection onto CG space is the same point *z* ∈ Ω̂. Following the exposition in ref 19, one can define the projected generator

$$L^{\xi }=\mathrm{PLP}$$ 10

which corresponds to the Markovian part in the Mori–Zwanzig decomposition. It turns out its action on a function ϕ = ϕ­(*z*) in CG space is given by

$$L^{\xi }(\phi )=P[L\xi ]\cdot \nabla_{z}\phi +\frac{1}{2}P[\nabla \xi^{T}a\nabla \xi ]:\nabla_{z}^{2}\phi$$ 11

As one can see, $L^{\xi }$ is of the same form as the original generator L in eq 6, and indeed it is the generator of an SDE *Z*_*t*_ on Ω̂

$$dZ_{t}=b^{\xi }\left(Z_{t}\right)dt+\sigma^{\xi }\left(Z_{t}\right)dW_{t}$$ 12

The effective drift and diffusion coefficients are given in analytical form by

$$\begin{array}{rl} b^{\xi }(z)=P(L\xi )(z) & a^{\xi }(z)=P(\nabla \xi^{T}a\nabla \xi )(z) \end{array}$$ 13

and the practical task of coarse graining is to approximate them numerically.

### 2.4 Generator EDMD

Numerical approximations to the infinitesimal generator L can be obtained by a data-driven learning method called generator extended dynamic mode decomposition^41^ (gEDMD). Given a finite set of scalar basis functions ψ­(*x*) = {ψ_1_(*x*),..., ψ_*n*_(*x*)}, and training data {*x*_*l*_}_*l* = 1_^*m*^ sampled from the invariant measure μ, we form the matrices

$$\begin{array}{rl} \Psi =[\psi_{i}(x_{l}){]}_{i,l}, & L\Psi =[L\psi_{i}(x_{l}){]}_{i,l} \end{array}$$

using the analytical formula ([Disp-formula eq6]) to evaluate the second of these matrices. The solution of a linear regression problem leads to the matrix approximation

$$\hat{L}={\hat{G}}^{-1}\hat{A}$$ 14

where

$$\begin{array}{ll} {\hat{A}}_{ij}=\frac{1}{m}\sum_{k=1}^{m}\psi_{i}(x_{k})L\psi_{j}(x_{k}), & {\hat{G}}_{ij}=\frac{1}{m}\sum_{k=1}^{m}\psi_{i}(x_{k})\psi_{j}(x_{k}) \end{array}$$ 15

These matrices are empirical estimators of the following mass, stiffness, and generator matrices

$$\begin{array}{lll} A_{ij}=\langle \psi_{i},L\psi_{j}\rangle_{\mu }, & G_{ij}=\langle \psi_{i},\psi_{j}\rangle_{\mu }, & L=G^{-1}A \end{array}$$ 16

The empirical mass matrix **Ĝ** is often ill-conditioned. A standard approach to circumvent this is to perform a whitening transformation based on removing small eigenvalues

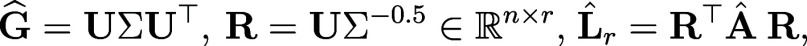

17
in which *r* ≤ *n*. Here, **R** is a transformation matrix mapping the original basis to the reduced basis

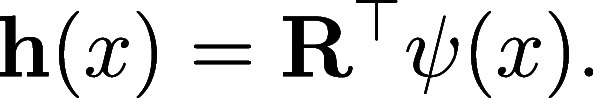

18
Dominant eigenvalues of the generator can be computed by diagonalizing the matrix **L̂** or **L̂**_*r*_.

For arbitrary stochastic dynamics, the computation of **A** involves a second-order differentiation as shown in eq 6. However, if the stochastic dynamics are reversible, only first-order derivatives are required to compute the matrix **A**, as the generator satisfies the following integration-by-parts formula

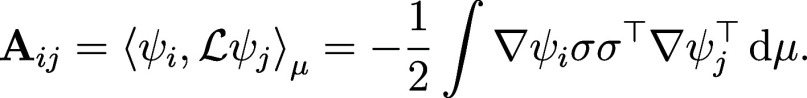

19
Importantly, if the basis functions are actually defined in a CG space Ω̂, that is ψ_*i*_(*x*) = ψ_*i*_(ξ­(*x*)), then by the chain rule the matrix **A** can be written as

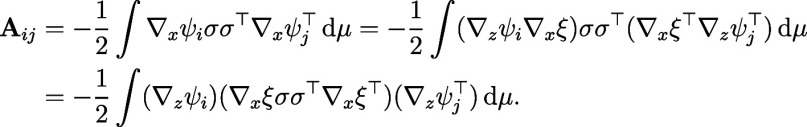

20
We refer to the matrix

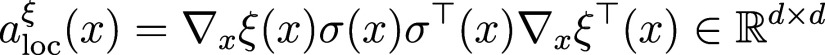

21
as *local diffusion*, and note that it is independent of the basis functions. It can therefore be computed a priori in numerical calculations.

### 2.5 Random Fourier Features

The gEDMD algorithm requires choosing a set of basis functions ψ­(*x*). In this work, we use random Fourier features (RFFs), which are defined as

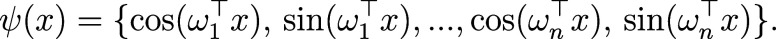

22
The vectors ω_1_,..., ω_*n*_ are random frequency vectors drawn from a spectral distribution ρ. RFFs provide a low-rank approximation to a reproducing kernel function,^42^ and can therefore generate a powerful basis without the need for manual basis set design. The precise relation between kernel-based gEDMD and random features was presented in ref 47. In the following applications, we use the spectral measure associated to a Gaussian squared exponential kernel with bandwidth parameter γ

$$k(x_{i},x_{j})=\exp\left(-\frac{∥x_{i}-x_{j}∥{)}^{2}}{2\gamma^{2}}\right)$$ 23

or to a periodic Gaussian kernel^48^ on periodic domains, such as dihedral coordinates.

## 3 Methods

We now turn to the suggested framework for learning CG dynamics based on the projection formalism and gEDMD models. We recall that the dynamical equation in CG space is given by ([Disp-formula eq12]), where the drift can be written as follows because of reversibility^43^

$$b^{\xi }=-\frac{1}{2}a^{\xi }\nabla_{z}F+\frac{1}{2}\nabla \cdot a^{\xi }$$ 24

### 3.1 Diffusion Learning

By eq 13, the analytical effective diffusion *a* ^ξ^ is the best-approximation of the local diffusion *a*_loc_^ξ^ by a (matrix-valued) function on the CG space. Hence, we can solve the following data-based minimization problem

$$a^{\xi }=\underset{a=a(z)}{\arg\;\min}\;\frac{1}{m}\sum_{i=1}^{m}∥a(\xi (x_{i}))-a_{\mathrm{loc}}^{\xi }(x_{i}){∥}_{F}^{2}$$ 25

where ∥·∥_*F*_ is the Frobenius norm for matrices. We parametrize the diffusion field *a* ^ξ^ element-wise as a linear combination of the reduced RFF basis

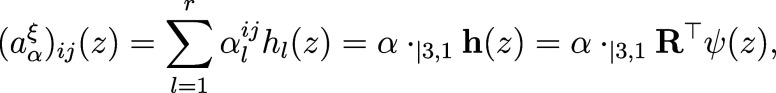

26
where we view the coefficient array α as a third-order tensor of dimension *d* × *d* × *r*, and the symbol ·_|*i*,*j*_ denotes contraction over indices *i* and *j* of two arrays. The parametrization must be symmetric, i.e., *a*_*ij*_^ξ^ = *a*_*ji*_^ξ^, and we may also choose to set specific elements to zero, for example to enforce a diagonal diffusion field. With the parametrization ([Disp-formula eq26]), the minimization problem ([Disp-formula eq25]) becomes a regression problem that can be directly solved, potentially after regularization. The complexity of the algorithm is governed by the cost of building **L̂**_*r*_ and learning the diffusion coefficients α. For a diagonal diffusion field, these costs can be estimated as $O(mdp^{2})$ and $O(mr^{2}+dmr)$ , respectively, where *m* is again the number of samples, *d* the dimension of the CG space, *p* is the number of random Fourier features, and *r* is the rank of **L̂**_*r*_. The critical parameters are therefore the number of random features *p* and the effective basis set size *r*.

### 3.2 Recovery of Spectral Properties

After solving the minimization problem ([Disp-formula eq25]), we can make use of the gEDMD method to assess the dynamical properties of the learned SDE in CG space. Using the integration-by-parts formula ([Disp-formula eq19]), the elements of the reduced generator matrix corresponding to the diffusion field ([Disp-formula eq26]) with coefficient array α are
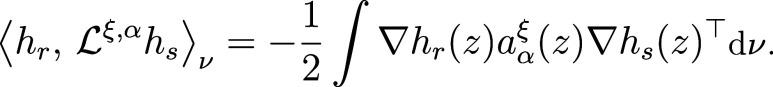


In matrix notation, this leads to the following explicit formula for the parametrized generator matrix, which can be computed directly without resorting to numerical simulations of the CG dynamics

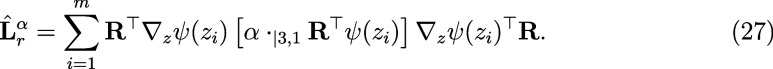

27
Properties inferred from the matrix **L̂**_*r*_^α^ can be compared to those obtained from the original gEDMD matrix **L̂**_*r*_ estimated off the full-space simulation data. For example, diagonalization of both **L̂**_*r*_ and **L̂**_*r*_^α^ leads to estimates λ_*i*_ and λ_*i*_^α^ for the dominant generator eigenvalues, which can be systematically compared. We mainly resort to comparing dominant eigenvalues in the examples below, but we point out that a more detailed assessment is possible: for instance, by computing matrix exponentials exp­(*t* **L̂**_*r*_) and exp­(*t* **L̂**_*r*_^α^), time-correlation functions can also be evaluated.

### 3.3 Learning the Effective Potential

We have seen that the accuracy of the effective diffusion field largely determines the dynamical properties of the coarse grained dynamics. In order to run simulations of the CG dynamics, and to ensure thermodynamic consistency, the effective potential *F* also must be learned in a parametric form. This is not the main focus of our study, hence we just point out a few options. A well-known and generally applicable technique is *force matching*,^24^ which is based on the following minimization problem for the effective force

$$\nabla_{z}F=\underset{g=g(z)}{\arg\;\min}\;\frac{1}{m}\sum_{i=1}^{m}∥g(\xi (x_{i}))-f_{\mathrm{lmf}}^{\xi }(x_{i}){∥}^{2}$$ 28

where *f*_lmf_^ξ^ is called *local mean force* and defined as follows

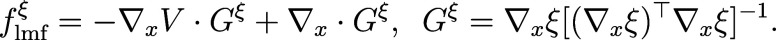

29
We point out the similarity to ([Disp-formula eq25]), which also led us to the name local diffusion for *a*_loc_^ξ^. The effective potential can be parametrized as a linear combination of basis functions, such as random features, or as a deep neural network.^13^ In low-dimensional CG spaces, it also possible to approximate the projected invariant distribution ν as a linear combination of kernel functions centered at the data sites, known as *kernel density estimate* (KDE).^49^ Since we only consider low-dimensional CG spaces here, we opt for the KDE option in the examples below.

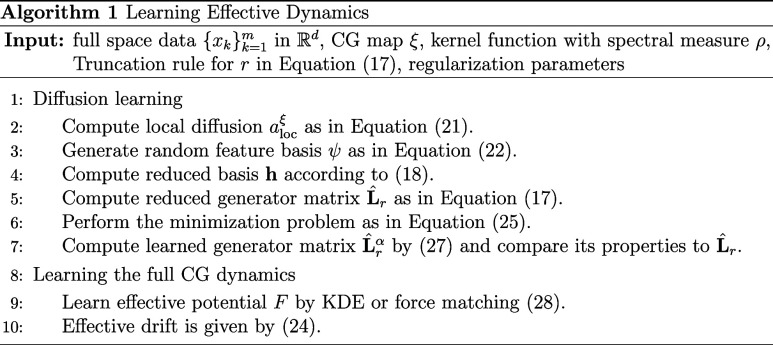



### 3.4 Overdamped Models for Molecular Systems

In practical MD simulations, computation of the local diffusion ([Disp-formula eq21]) requires knowledge of the full-state diffusion tensor, which depends on the thermostat used to drive the molecular dynamics. If the full-state dynamics are just overdamped Langevin dynamics ([Disp-formula eq3]), the local diffusion reduces to the following simple form:

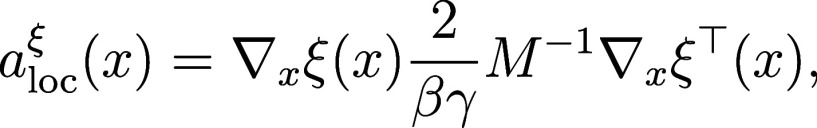

30
where *M* is the diagonal mass matrix of all atoms.

Very often, however, one can apply an overdamped approximation. If the full-state dynamics are underdamped Langevin, then averaging theory^50^ shows that for small friction γ, and under a rescaling of time, the position space dynamics are close to the overdamped process ([Disp-formula eq3]). In practice, we observe empirically that even if the friction is not asymptotically small, and even for thermostats different from underdamped Langevin, one can find a rescaling of time such that the position process is similar to an overdamped process.

We therefore apply the overdamped approximation of the local diffusion ([Disp-formula eq30]) in the molecular examples in Sections 4.2 and 4.3. This simplification is also convenient as a mature theory of the projection formalism for underdamped dynamics is still under construction, see ref 51 for some preliminary results.

As the overdamped approximation is expected to hold after a rescaling of time, the time scales of the resulting CG model will be faster than those of the original dynamics. To account for this rescaling, we can make use of the existing simulation data to also compute a standard kinetic model for the Koopman operator, for example a Markov state model **T** ^*t*^ at a suitable lag time *t* > 0. It is sufficient to construct the MSM in CG space, hence the definition of appropriate MSM states is not challenging. By comparing the MSM time scales to those of the learned generator **L̂**_*r*_^α^, the rescaling of time can be practically computed.

## 4 Examples

To show the effectiveness of the proposed method, we apply it to a two-dimensional model system defined by the Lemon-slice potential, and to MD simulation data of the alanine dipeptide and of the mini-protein Chignolin, which are widely used test cases in molecular dynamics.

### 4.1 Lemon-Slice Potential

#### 4.1.1 System Introduction

The Lemon-slice system is governed by overdamped Langevin dynamics in eq 3 with the following potential *V*

$$V(x,y)=V(r,\phi )=\cos(4\phi )+10(r-1{)}^{2}$$ 31

where *r* and ϕ are polar coordinates. The energy landscape of the system is shown in Figure 1a. To form the SDE for this example, we consider a diagonal state-dependent diffusion field σ­(*x*) defined as

$$\sigma (x)=\left[\begin{array}{cc} \sqrt{\frac{2}{\beta }(\sin(\phi )+1.5)} & 0\\ 0 & \sqrt{\frac{2}{\beta }(\sin(\phi )+1.5)} \end{array}\right]$$ 32

where β = 1 is the inverse temperature. Using the Euler–Maruyama scheme at discrete integration time step *dt* = 10^–3^ for integration of the SDE, we collect the training data for learning. For the sake of validation and showing the robustness of the method, we produce 5 independent experiments, each with length of *m* = 10^5^ time steps. We further down sample them to 1000 samples each for learning effective force and diffusion.

**Figure 1:**
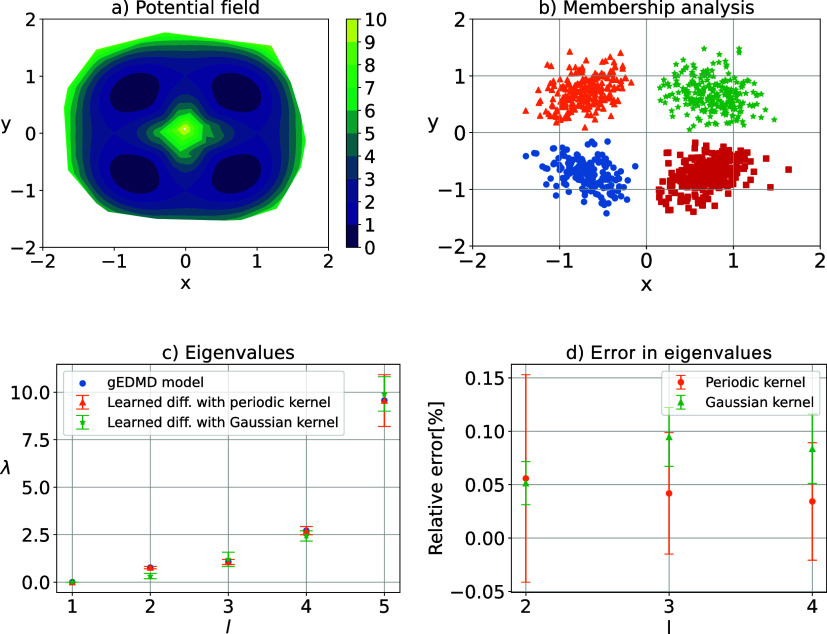
Approximation of generator for the Lemon slice system. Potential field in (a). Membership analysis in (b) using 1000 samples. The dominant eigenvalues of the reference generator **L̂**_*r*_ and the learned generator **L̂**_α_^ξ^ built upon the learned effective diffusion, using Gaussian and periodic Gaussian kernels, in (c). The relative error of these eigenvalues compared to the reference is shown in (d).

As shown in previous studies,^20^ the polar angle ϕ is a suitable CG coordinate for this system, as it resolves all four metastable states

ξ(x,y)=ϕ 33

For this system, analytical expressions for the effective drift and diffusion along ξ can be obtained by a slight modification of the results in ref 20, and serve as reference values.

We apply our learning method with random Fourier features on the reaction coordinate ξ, to identify the generator eigenvalues and metastable states and, subsequently, to identify an effective dynamics along ξ using Algorithm 1. As the polar angle is a periodic reaction coordinate (RC), we use the spectral measures associated to both a periodic and nonperiod Gaussian kernel and compare them. The number of random features and the kernel bandwidth in either versions of Gaussian kernel are optimized using cross validation based on the VAMP-score.^39^ Details on the VAMP-score analysis are reported in the Supporting Information.

#### 4.1.2 Meta-Stability Analysis

Figure 1c shows the leading eigenvalues obtained from the generator matrix **L̂**_*r*_. As one notices, there are four dominant eigenvalues followed by a gap. These four eigenvalues are corresponding to the four minima in the potential field. Having determined the eigenvectors of the generator, we can perform robust Perron Cluster Cluster Analysis (PCCA+)^52^ algorithm to assign to each sample point its membership to each metastable state. Figure 1b shows that the four potential minima are perfectly recovered in this way. A comparison of the leading eigenvalues of the reference model **L̂**_*r*_ and the learned matrix **L̂**_α_^ξ^ for the optimal parameters α is shown in Figure 1c. Both choices of the kernel function lead to satisfactory results, the periodic kernel provides slightly higher accuracy in approximation of the generator eigenvalues. Note that the kernel bandwidth is tuned for each kernel function separately.

#### 4.1.3 Analysis of the CG Dynamics

The learned generator providing the eigenvalues reported above is built upon the effective diffusion shown in Figure 2b, which is almost perfectly following the reference. Furthermore, we perform the force matching as well and obtain the effective force in the CG space shown in Figure 2a. From the effective force and diffusion, the effective drift can be obtained according to eq 24, which is also compared against the analytical expression in Figure 2c, likewise showing very good agreement.

**Figure 2:**
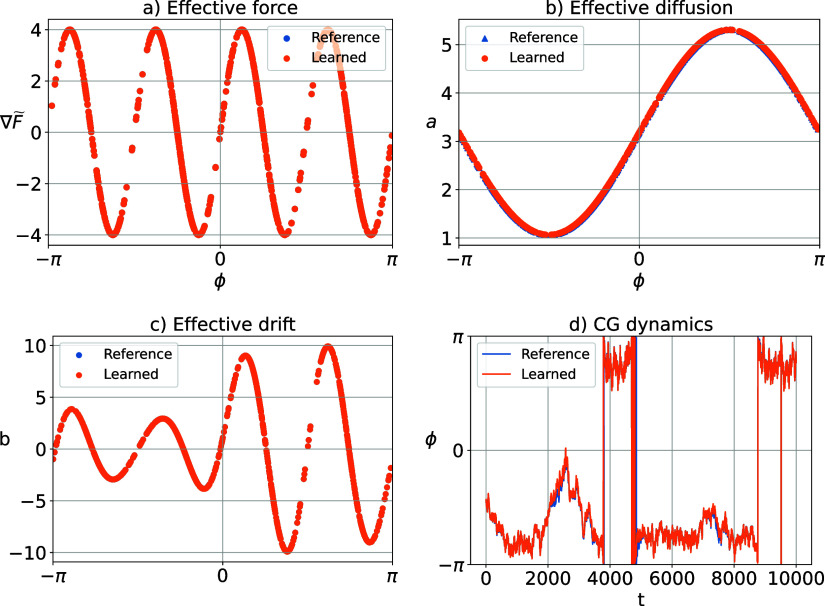
Application of Algorithm 1 to identify angular dynamics for the Lemon-slice system. Effective force in (a), effective diffusion in (b), effective drift in (c), and integration of an example trajectory, using both the reference and learned SDE in (d).

With the effective drift and diffusion fields, we are able to simulate the learned SDE governing the CG coordinate. We use the Euler-Maruyama scheme to integrate the learned and reference SDEs with integration time step of *dt* = 10^–3^. Figure 2d shows two trajectories of the CG coordinate ϕ for both dynamics for 10^4^ time steps, using the same Brownian motion for both trajectories. The propagated learned system follows the reference closely, with both systems staying long times in each metastable state, and rarely swapping in between those. Combined, the results above demonstrate that the proposed method can approximate the full system’s metastable sets well, and identify a suitable SDE for CG dynamics which is accurate even on the level of individual trajectories.

As a final analysis, we compare the properties of the learned CG model with variable diffusion to those of a CG dynamics with constant diffusion, in order to demonstrate the necessity of allowing a state-dependent diffusion. We set the effective diffusion for the constant model to $a=\frac{2}{\beta }=2$ . We propagate the corresponding SDEs for a sufficiently large span of time, and estimate a new generator EDMD model based on these simulations. Figure 3, shows the eigenvalues of the generator for these cases compared to the learned generator built upon the original data set. The result shows that learning a state-dependent diffusion is necessary to recover the original system’s leading eigenvalues.

**Figure 3:**
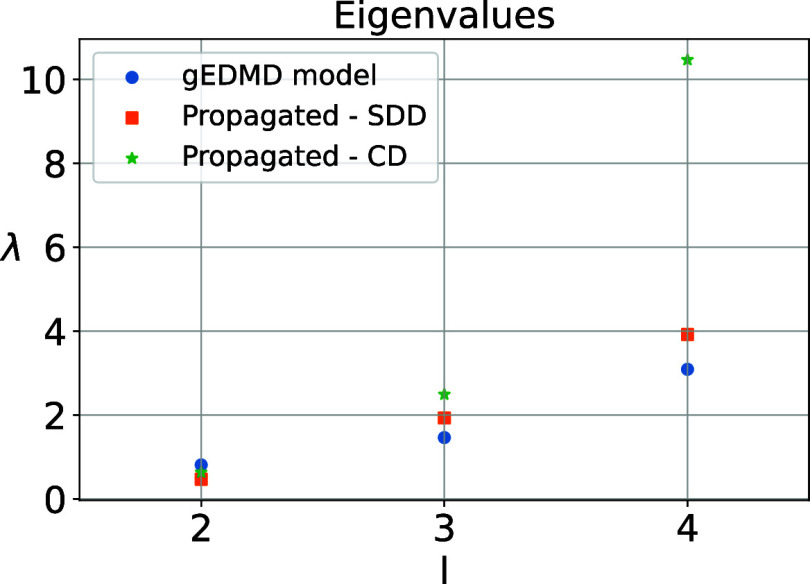
Dominant eigenvalues of the generator, using models built on simulation data of the learned coarse grained dynamics with state-dependent diffusion (SDD, orange) and with constant diffusion (CD, green). As a comparison, we show the eigenvalues of the generator **L̂**_*r*_ using the original data set (blue). Note that the first eigenvalue is omitted as it is zero.

### 4.2 Alanine Dipeptide

#### 4.2.1 System Introduction

Alanine dipeptide is a model system widely used in method development for simulation studies of macro-molecules. Figure 4 shows the graphical representation of Alanine dipeptide. It is well-known that the dynamical behavior of the molecule can be expressed in terms of the backbone dihedral angles ϕ and ψ, which constitute the two-dimensional reaction coordinate space defining the CG map ξ:

$$\xi (x)=[\begin{array}{ll} \phi (x) & \psi (x) \end{array}]$$ 34

We generated a 500 ns simulation of the system in explicit water, the details of the simulation settings are summarized in the Supporting Information.

**Figure 4:**
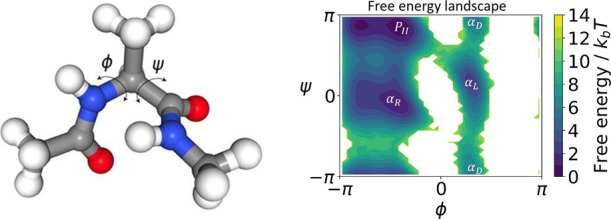
Graphical representation of the alanine dipeptide molecule on the left, and the reference free energy profile in two-dimensional dihedral angle space on the right.

The familiar free energy landscape of the system with respect to these two angles is shown in Figure 4, displaying four minima, two on the left side, usually denoted (*P*_II_, α_R_), and two in the central part, called (α_D_, α_L_).

We apply the gEDMD algorithm with random Fourier features to find the metastable sets, and then use Algorithm 1 to learn the effective force and a state-dependent effective diffusion field in the dihedral angle space. Because of the periodicity of the CG coordinates, ϕ and ψ, the spectral measure corresponds to a periodic Gaussian kernel. Similar to the previous example, we tune the bandwidth of the kernel function as well as the size of random features using the VAMP-score.

#### 4.2.2 Meta-Stability Analysis

Figure 5a shows the leading finite time scales by taking reciprocals of the first three nonzero eigenvalues of the generator obtained from the gEDMD matrix **L̂**_*r*_ (error bars in the figure are generated by analyzing 5 independent subsampled sets of the original data set, each comprising 50,000 samples). The figure indicates the three dominant time scales which are corresponding to the four minima in the free energy landscape followed by a gap. In addition, we also show the time scales corresponding to the generator **L**_α_^ξ^ based on the optimal effective diffusion, which agree well with the reference. Note that the generator time scales shown have been rescaled after comparison to a Markov state model **T** ^*t*^ trained on the original simulation data, as described in Section 3.4. This comparison showed that the time scales of the generator models **L̂**_*r*_ and **L̂**_α_^ξ^ were smaller than those of the MSM model by a uniform factor of about 100, meaning that the dynamics in CG space based on the overdamped assumption is accelerated by a factor 100 for this example. After applying the uniform rescaling, the generator time scales match those of the MSM analysis very well.

**Figure 5:**
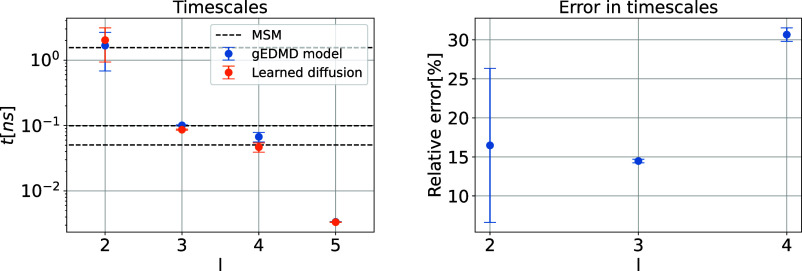
Approximation of generator for alanine dipeptide. The dominant time scales corresponding to the reference generator **L̂**_*r*_ and the learned generator **L̂**_α_^ξ^ built upon the learned effective diffusion on the left, and the relative error of these time scales on the right. The time scales of the MSM model are shown as black dashed lines for comparison. Note that time scales of the generators are rescaled by a factor of 100 to account for the overdamped approximation. The first time scale (*l* = 2) corresponds to the transition between the left-hand side and the central part, the second one (*l* = 3) corresponds to the transition between *P*_II_ and α_*R*_, and the third one (*l* = 4) corresponds to the transition between α_D_ and α_L_.

#### 4.2.3 Analysis of the CG Dynamics

For this 2-dimensional coarse graining, we can express the diffusion field as a 2 × 2 full matrix. For simplicity, however, we assume that the learned diffusion is a diagonal matrix. Figure 6 shows the first and second diagonal terms of the learned diffusion field based on 50000 samples of the available data set. To learn the effective potential, we found that the KDE method works best. The reference and learned effective free energy surfaces are depicted in Figure 6c,d, respectively. It it noticeable that the learned free energy surface correctly captures all energetic minima and barriers up to some minor spurious behavior close to the transition regions. We emphasize once again that this approximation could probably be improved further by using a more accurate learning method.

**Figure 6:**
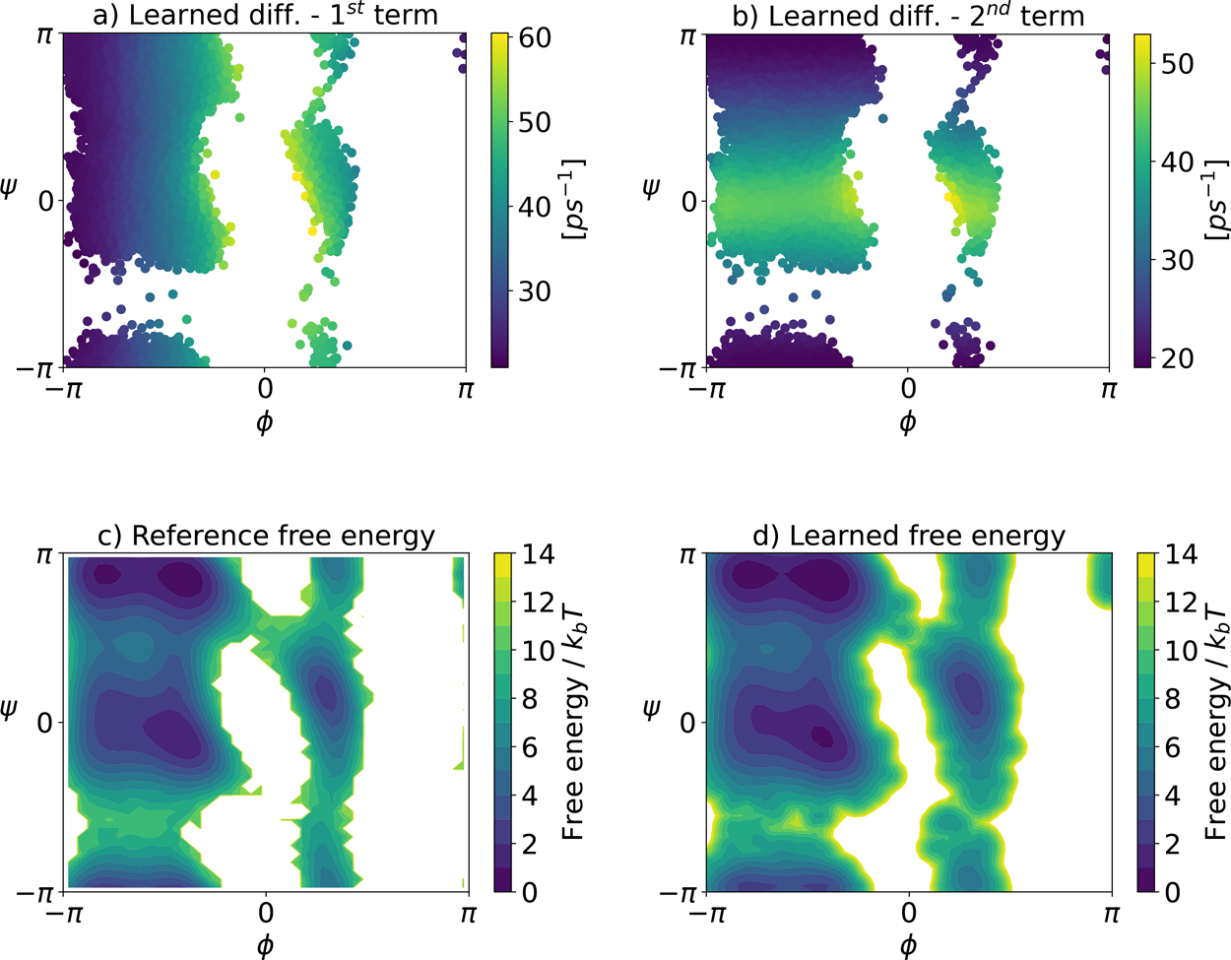
First (a) and second (b) diagonal terms of the learned diffusion covariance matrix, the reference free energy surface (c) and the free energy surface learned via KDE (d).

From the effective force and diffusion, one can compute the effective drift from which the SDE governing the dynamics in the CG space can be formed. We integrate the learned SDE for 5 × 10^5^ integration steps, with an effective (rescaled) time step of 0.1 ps, corresponding to an effective total simulation time of 50 ns. Figure 7b shows the estimated free energy surface obtained from a histogram of the propagated data set which is somewhat less accurate than the learned potential. Since we are mainly interested in kinetic properties, we estimate a new gEDMD model on the propagated data set for the CG dynamics. We find that the four metastable states are correctly reproduced by a PCCA+ analysis of the propagated coarse grained SDE, as shown in the left panel of Figure 8. In addition, we show the resulting transition time scales on the right of Figure 8, compared to the ones corresponding to the learned generator built upon the original data set, as well as the rescaled MSM time scales. The results confirm that the two-dimensional CG dynamics with learned effective diffusion accurately recover the metastable states and transition time scales of the original dynamics, while adequately recovering their thermodynamic properties.

**Figure 7:**
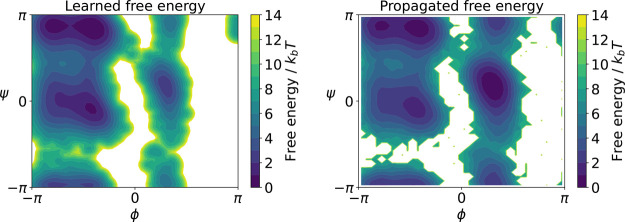
Left: Free energy surface learned via KDE. Right: estimated free energy surface from histogramming the simulated CG dynamics.

**Figure 8:**
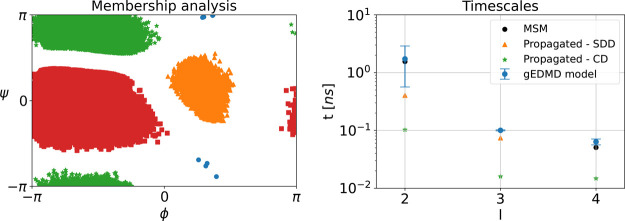
Kinetic consistency of the CG dynamics for alanine dipeptide. Left: PCCA+ membership analysis applied to simulation data of the CG dynamics. Right: slowest finite time scales calculated using an approximation of the generator from the reference data set (blue) and the propagated CG dynamics with state-dependent diffusion (SDD, orange) as well as constant diffusion (CD, green), compared to those obtained via a Markov state model (black).

As a final analysis, we also generate a trajectory of the coarse grained SDE, but with the diffusion set to a constant. We choose the value of constant diffusion according to the average of the learned diffusion on the original data set, resulting in *a* ≈ 30.25 ps^–1^. We also estimate a gEDMD model for these dynamics, and report the transition time scales in Figure 8. The result shows the necessity of learning a state-dependent diffusion field.

### 4.3 Chignolin

#### 4.3.1 System Introduction

Finally, we apply the proposed method to the *”*025*”* mutant of Chignolin (CLN025),^53^ which is a mini-protein consisting of 10 amino acids. Figure 9 shows the graphical representation of the molecule. The data for this example was obtained via simulation in *OpenMM* based on *AMBER99 SB-ILDN* force field, see ref 54 for details of the setup. The data set consists of 20 independent trajectories each for 5 μs.

**Figure 9:**
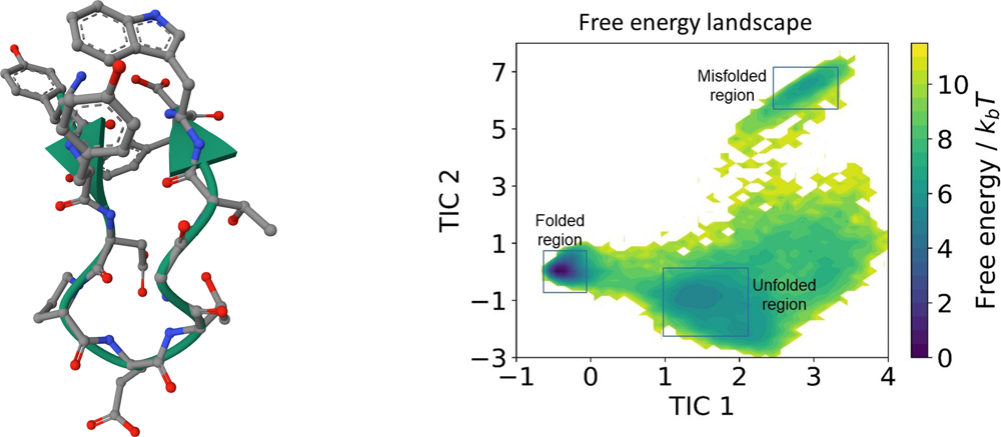
Graphical representation of CLN025 on the left,[Fn fn1] and the reference free energy surface in the two-dimensional TICA space on the right. The left-hand side minimum corresponds to the folded state, the bottom right minimum corresponds to the unfolded state and the top one associates to the misfolded state.

For this example, we need to find a coarse graining function in a data-driven manner. To obtain the CG space, we start with a 45-dimensional feature space comprising the *C* ^α^ distances of all residues. A straightforward linear method to find the CG coordinates is Time-Lagged Independent Component Analysis (TICA).^55^ As a result of TICA, we select the first 2 dominant components to constitute the RC space:

$$\xi \left(x\right)=\left[\begin{array}{cc} {\mathrm{TIC}}_{1}\left(x\right) & {\mathrm{TIC}}_{2}\left(x\right) \end{array}\right]$$ 35

By projecting the atomistic positional information of the system onto this 2-dimensional TICA space and computing the histogram of the data, the free energy surface can be obtained, as shown in Figure 9. As shown in previous studies, the two-dimensional TICA space adequately captures the slow dynamics. In particular, the free energy surface shows three minima, representing the three conformational states of folded, unfolded and misfolded.

#### 4.3.2 Meta-Stability Analysis

To find the time scales of the system, we applied the gEDMD method with random Fourier features as before, and computed the eigenvalues of the generator model **L̂**_*r*_. We performed the same analysis as for the previous example to tune the kernel bandwidth and the number of random features based on the VAMP-score, see the Supporting Information for details. Figure 10 shows the corresponding time scales of the system, which are the inverse of the generator’s eigenvalues. The figure indicates the two leading time scales of the system corresponding to the three metastable sets, followed by a spectral gap. Moreover, we show that the time scales of the CG generator **L**_α_^ξ^ for the optimal effective diffusion are very similar, the relative errors shown on the right of the same figure are sufficiently small. Also, we observe that the gEDMD time scales are once again uniformly rescaled compared to the leading time scales of an MSM estimated on the original data, see the previous example and Section 3.4. The rescaling factor is quite drastic this time, reducing microsecond time scales of the full system to less than pico-seconds for the CG dynamics. Nevertheless, as the rescaling is again uniform, the original time scales can be recovered by rescaling time. Error-bar figures were again generated by analyzing 5 independent subsampled sets, each comprising 1.6 × 10^5^ samples.

**Figure 10:**
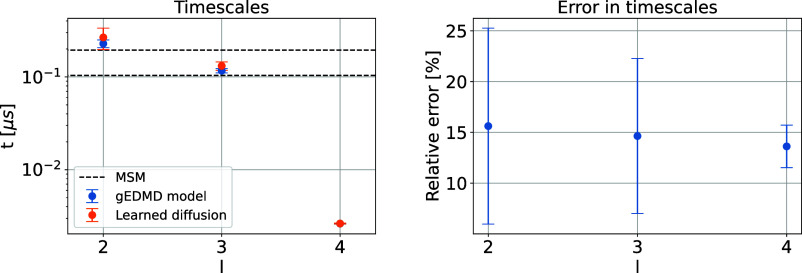
Approximation of generator for Chignolin. The slowest finite time scales corresponding to the reference generator **L̂**_*r*_ and the learned generator **L̂**_α_^ξ^ built upon the learned effective diffusion on the left, and the relative error on the right. The time scales of the MSM model on the original simulation data are shown as black dashed lines for comparison. Note that time scales of the generators are rescaled by a factor of 10^6^. The first time scale (*l* = 2) corresponds to the folded-unfolded transition and the second one (*l* = 3) corresponds to the unfolded-misfolded transition.

#### 4.3.3 Analysis of the CG Dynamics

Following the same procedure as in the previous examples, we learned a 2 × 2 diffusion matrix in the CG space, but this time, we tested out a full nondiagonal diffusion field. Figure 11 shows the four elements of the learned diffusion matrix. In addition, the left panel of Figure 12 depicts the free energy surface learned by the KDE method, which is in satisfactory agreement with the reference one in Figure 9.

**Figure 11:**
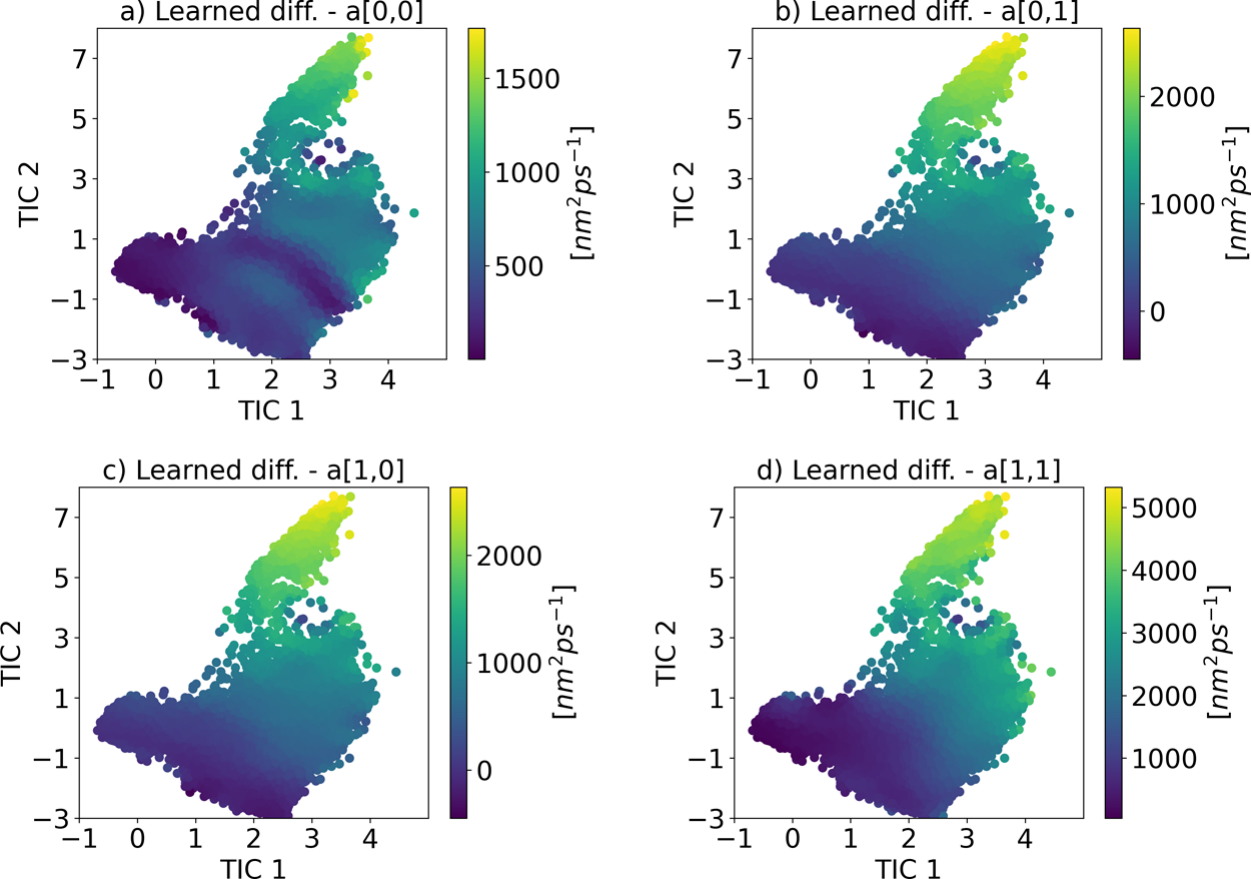
(a–d) Components of the learned diffusion covariance matrix for Chignolin in its two-dimensional TICA space (note that the off-diagonal elements are symmetric).

**Figure 12:**
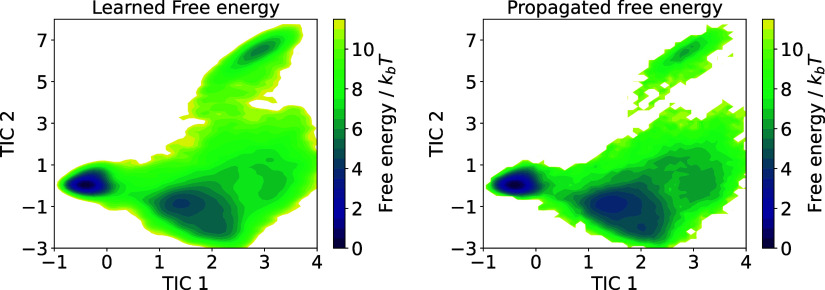
Free energy surface in the two-dimensional TICA space for Chignolin, as learned by the KDE estimator on the left, and obtained from a histogram of the CG dynamics on the right.

From the effective diffusion and potential energy, we compute the effective drift according to eq 24. We integrate the learned SDE for 5 × 10^5^ integration steps, with an effective (rescaled) time step of *dt* = 20 ps, corresponding to an effective total simulation time of 10 μs. The right panel in Figure 12 shows the estimated free energy surface obtained from a histogram of the propagated CG dynamics. Once again, we find it in satisfactory agreement with the learned and the reference free energy in the CG space. Its accuracy could likely be improved by applying a more accurate learning method.

As we are mainly interested in kinetic properties, we compute a new gEDMD model on the propagated CG dynamics, and recompute the associated eigenvalues and eigenvectors. The result of a PCCA+ analysis indicates that the correct metastable sets are recovered, as shown in the left panel in Figure 13. Likewise, the leading implied time scales estimated from the simulated CG dynamics are in good agreement with those of the original gEDMD model **L̂**_*r*_ and the rescaled MSM time scales, both estimated from the original simulation data, as shown in the right panel of Figure 13.

**Figure 13:**
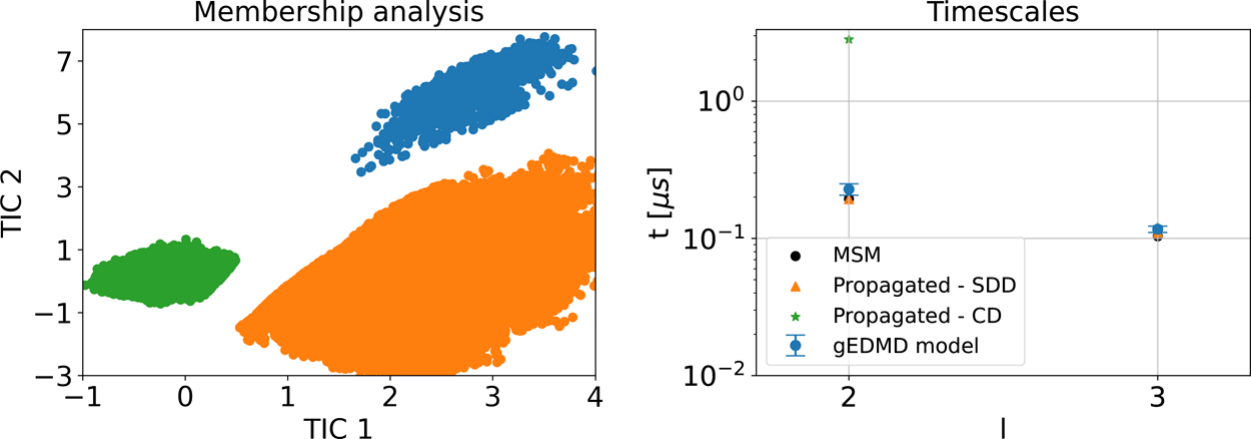
Kinetic consistency of the learned CG model for Chignolin. Left: PCCA+ states obtained from simulating the learned CG model. Right: Slowest finite time scales of the system calculated using an approximation of the generator from the reference data set (blue) and from the propagated CG dynamics (state-dependent diffusion in orange, constant diffusion in green). We also compare to rescaled time scales from a Markov state model on the original simulation data (black).

Similar to the previous example, we also generate a separate trajectory based on a constant diffusion according to the average of the learned diffusion. We find that transition time scales for the constant diffusion are not well fitted to the reference. Due to taking the average, too much detailed information about the diffusion field is lost, leading to different time scales. This result confirms the need to learn a state-dependent diffusion field in the CG space to achieve kinetic consistency.

## 5 Discussion

We presented a novel approach to learn kinetically consistent coarse grained models for stochastic dynamics. We have introduced a learning method for the effective diffusion field in CG space, and shown how the kinetic properties of the CG dynamics can be evaluated by exploiting models for the Koopman generator (gEDMD algorithm). We have also shown that random Fourier features provide an efficient and flexible parametrization for both the effective diffusion and the gEDMD model. By means of three examples, a two-dimensional model potential and two data sets of molecular dynamics simulations, we showed that the effective dynamics in low-dimensional reaction coordinate spaces are able to reproduce both thermodynamic and kinetic quantities of the full dynamics accurately.

For the molecular examples, we have relied on the overdamped assumption to parametrize reversible CG dynamics. We have seen that this assumption leads to a uniform acceleration of the CG dynamics compared to the full system. The rescaling factor can be estimated numerically by comparing the gEDMD model to a kinetic model that does not rely on the overdamped assumption. We used MSMs in this paper, but note that a more general EDMD model (e.g., using random features) would work just as well.

In this study, we used long equilibrium simulations to train CG models. However, one of the appealing aspects of the generator EDMD approach is that it only requires Boltzmann samples. As has been pointed out in previous studies, these samples can also be obtained from biased sampling simulations,^56,57^ or by employing generative models.^58^

Among other topics, future work will focus on applying the formalism to higher-dimensional and more transferrable CG coordinates, for example C-alpha models. We do not anticipate a principal limitation to applying our method in higher-dimensional spaces. Learning the effective diffusion and the gEDMD model, which is crucial to validate kinetic consistency of the CG model, might require more careful parameter choices in higher-dimensional spaces. This is currently under investigation. Another topic is the construction of CG models that can explicitly account for the underdamped structure of the full system, or that can incorporate memory terms, which were entirely disregarded in our study. Moreover, one can also try to simultaneously optimize the CG mapping ξ along with the parameters of the CG model, for instance by balancing the VAMP score versus the complexity of the CG model.

## Supplementary Material



## Data Availability

Codes and data to reproduce the results and figures shown in this manuscript are available from the following public repository: 10.5281/zenodo.15209618.

## Footnotes

1 The image is generated using Protein Data Bank in Europe platform.

## Notes

VAMP-score; simulation settings for alanine dipeptide; references; and additional figures and tables (PDF)
