# Supplementary material for: Kinetically Consistent Coarse Graining Using Kernel-Based Extended Dynamic Mode Decomposition
Source: J Chem Theory Comput. 2025 Jul 18;21(15):7236–48. doi: 10.1021/acs.jctc.5c00479 (PMC12355707; doi:10.1021/acs.jctc.5c00479)
Supplement: Supplementary file 1 [file ct5c00479_si_001.pdf]

Kinetically Consistent Coarse Graining using Kernel-based Extended Dynamic Mode Decomposition
Supporting Information

Vahid Nateghi and Feliks Nüske*

Max-Planck-Institute for Dynamics of Complex Technical Systems, Magdeburg 39106, Germany

E-mail: nateghi@mpi-magdeburg.mpg.de,nueske@mpi-magdeburg.mpg.de

1 VAMP-score

We tune hyper-parameters of the proposed method based on *VAMP variational principle* proposed in Ref.[1] , stating that for reversible systems, the k dominant eigenvalues of the Koopman generator can be obtained by a minimization problem

$$\sum_{i=1}^{k} \lambda_i = \min_{\phi_0,\ldots,\phi_k} \sum_{i=1}^{k} \langle \phi_i, \mathcal{L}\phi_i \rangle_\mu \tag{1}$$

where the ϕ_i are orthogonal functions. We use this variational principle to optimize the kernel bandwidth giving rise to the spectral measure used in our proposed method, and to perform sensitivity analysis on the random Fourier feature size. To do this robustly and avoid overfitting, we make use of standard cross validation scheme by introducing 40% of dataset as test set.

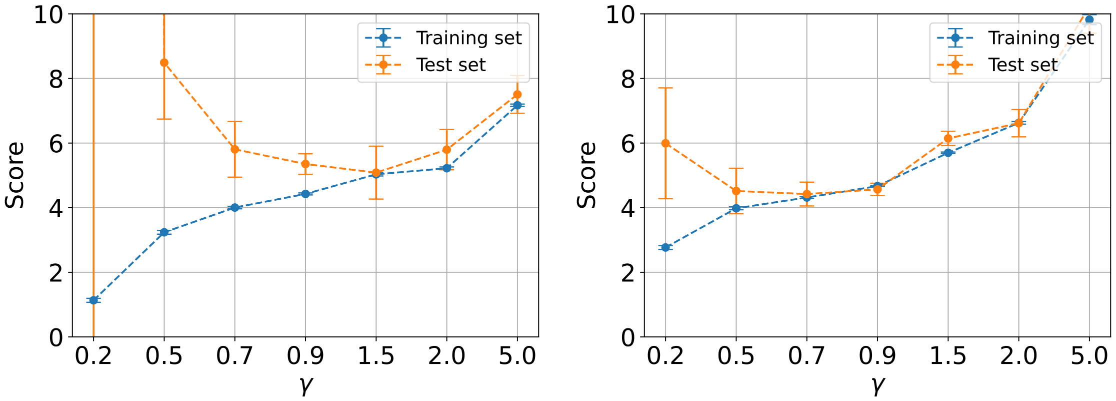

Figure 1: VAMP-score analysis for the Lemon slice example using periodic Gaussian kernel on the left and Gaussian kernel on the right. We considered $\gamma = 1.5$ and $\gamma = 0.7$ for the case with periodic Gaussian and Gaussian kernels, respectively.

Figure 1 shows the result of optimizing the bandwidth for the lemon slice example, using Gaussian and periodic Gaussian kernels. We applied the same procedure to alanine dipeptide and Chignolin. Figure 2 shows the result for optimization of the bandwidth for the molecular systems.

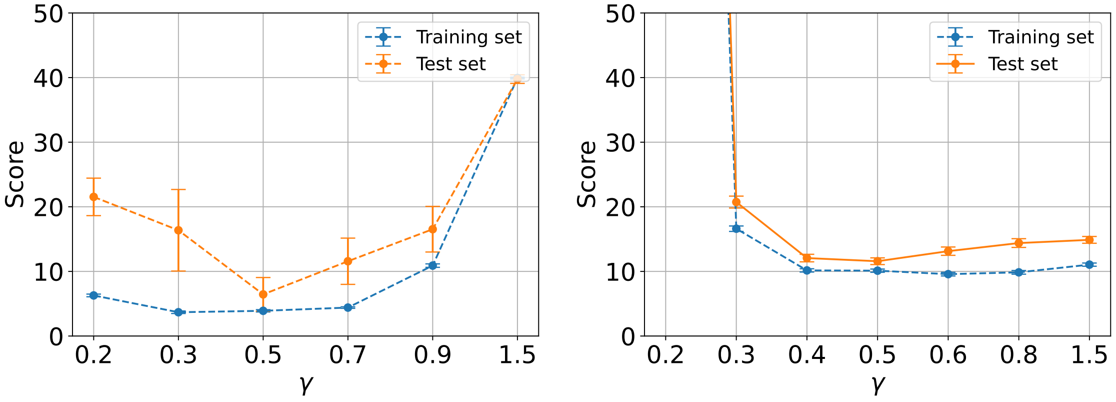

Figure 2: VAMP-score analysis for alanine dipeptide on the left and Chignolin on the right. We considered $\gamma = 0.5$ and $\gamma = 1$ for alanine dipeptide and Chignolin experiments, respectively.

As the figure indicates, there exists a range of the bandwidth γ that we can safely choose the bandwidth from. Moreover, we can repeat the experiment by testing different values

for the number of random Fourier features p. Figure 3 summarizes the sensitivity of the VAMP-score to the feature size.

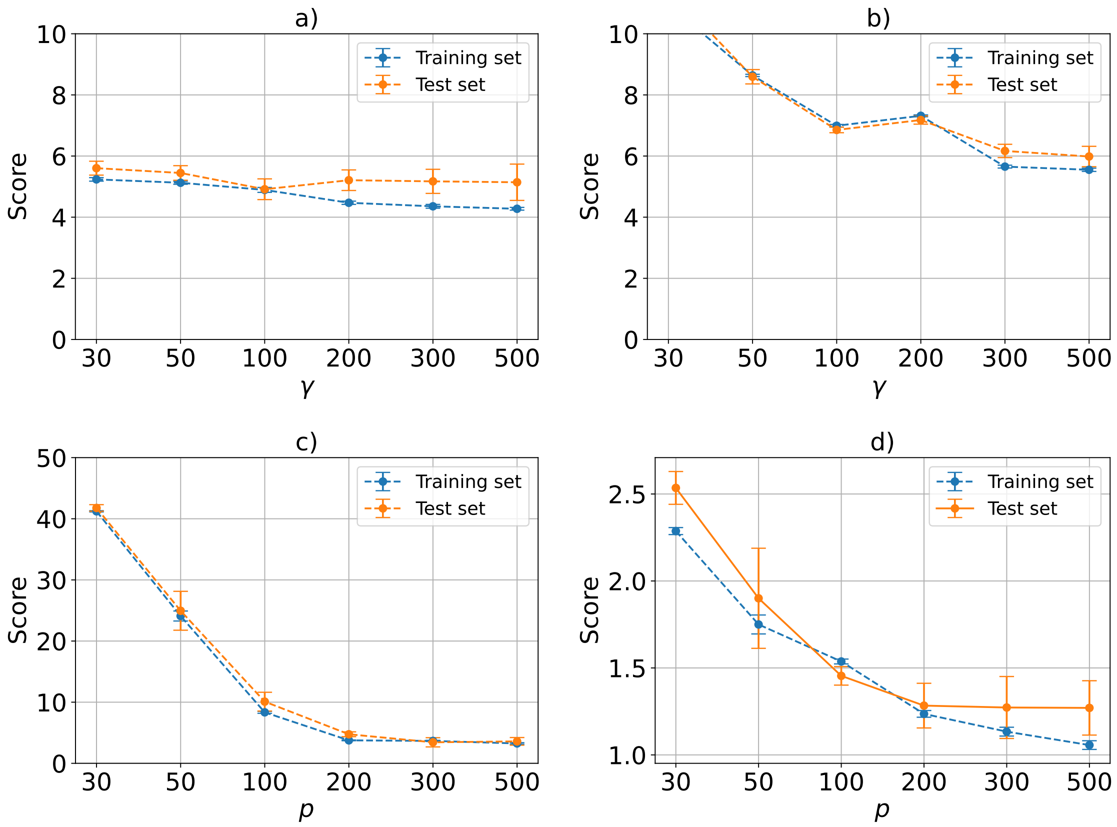

Figure 3: Sensitivity analysis of the VAMP-score for random Fourier feature size. Analysis for Lemon slice potential based on Gaussian and periodic Gaussian kernels in (a) and (b), alanine dipeptide in (c) and Chignolin in (d). In our experiments, we considered $p = 50$, $p = 300$, $p = 100$ for Lemon slice, alanine dipeptide, and Chignolin, respectively.

Notably, the score remains constant across a range of feature sizes. To reduce computational cost and avoid an overfitting, we use the smallest feature size from this range. Moreover, we depict the rank of the reduced generator based on different values for the number of Fourier features in Figure 4.

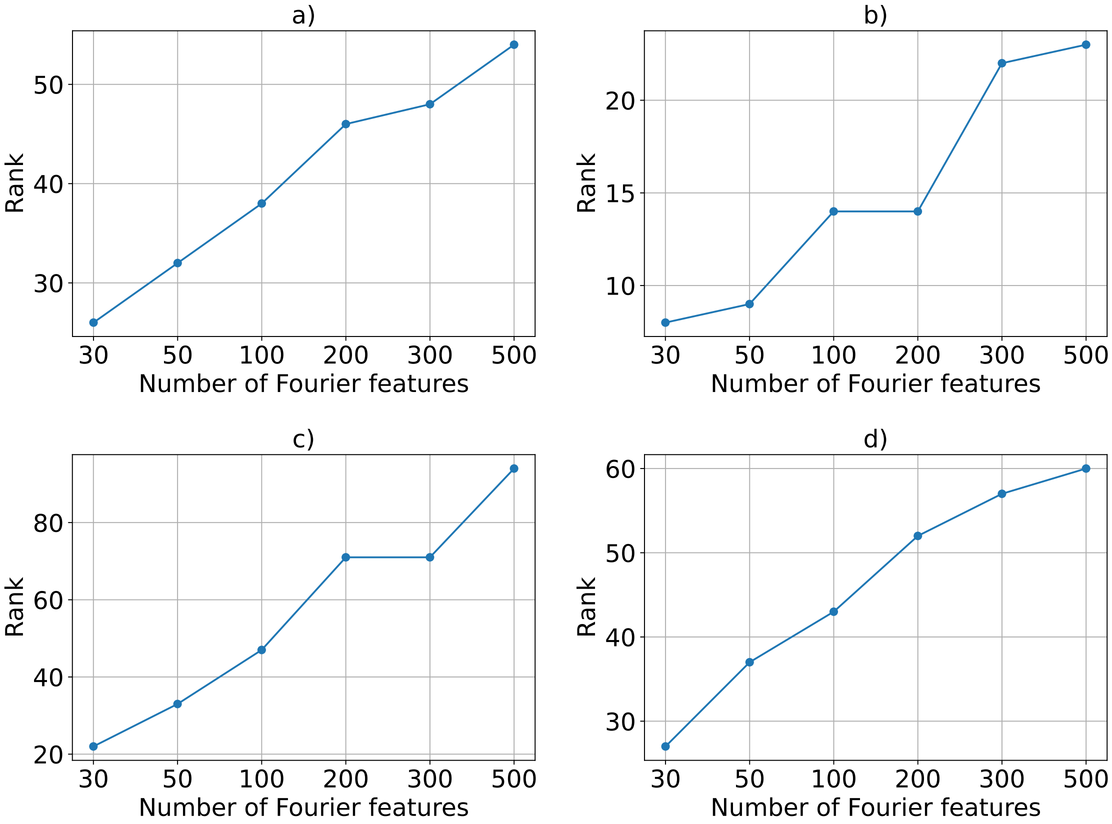

Figure 4: Sensitivity of the generator's rank to the number of random Fourier features. Analysis for Lemon slice potential based on Gaussian and periodic kernel in (a) and (b), respectively, alanine dipeptide in (c), and Chignolin in (d).

2 Simulation Settings for Alanine Dipeptide

For the example of alanine dipeptide, we used the Gromacs[2] simulation software to produce a 500 ns simulation. The details of the input setting we used for running the simulation is summarized in Table 1.

Table 1: Experiment setup

Force Field	AMBER99SB-ILDN
Temperature	300 K
Time constant ($1/\gamma$)	0.2 ps
Integrator	Langevin dynamics
Time step	2 fs
Simulation time	500 ns
Export data frequency	100 fs

References

(1) Wu, H.; Noé, F. Variational approach for learning Markov processes from time series data. *Journal of Nonlinear Science* **2020**, *30*, 23–66.

(2) Bekker, H.; Berendsen, H.; Dijkstra, E.; Achterop, S.; Vondrumen, R. v.; Vanderspoel, D.; Sijbers, A.; Keegstra, H.; Renardus, M. Gromacs-a parallel computer for molecular-dynamics simulations. 4th international conference on computational physics (PC 92). 1993; pp 252–256.
